# Quantitative Assessment of Chirality of Protein Secondary Structures and Phenylalanine Peptide Nanotubes

**DOI:** 10.3390/nano11123299

**Published:** 2021-12-05

**Authors:** Alla Sidorova, Vladimir Bystrov, Aleksey Lutsenko, Denis Shpigun, Ekaterina Belova, Ilya Likhachev

**Affiliations:** 1 Faculty of Physics, Lomonosov Moscow State University, 119991 Moscow, Russia; aleksluchrus@yandex.ru (A.L.); denish.den@mail.ru (D.S.); ev.malyshko@physics.msu.ru (E.B.); 2 Institute of Mathematical Problems of Biology, The Branch of Keldysh Institute of Applied Mathematics, RAS, 142290 Pushchino, Russia; vsbys@mail.ru (V.B.); ilya_lihachev@mail.ru (I.L.)

**Keywords:** helical structures, peptide nanotubes, phenylalanine, self-assembly, molecular modeling, dipole moments, polarization, chirality, protein secondary structure

## Abstract

In this study we consider the features of spatial-structure formation in proteins and their application in bioengineering. Methods for the quantitative assessment of the chirality of regular helical and irregular structures of proteins are presented. The features of self-assembly of phenylalanine (F) into peptide nanotubes (PNT), which form helices of different chirality, are also analyzed. A method is proposed for calculating the magnitude and sign of the chirality of helix-like peptide nanotubes using a sequence of vectors for the dipole moments of individual peptides.

## 1. Introduction

There exists a sophisticated understanding of the relationship between amino acid sequences and the structure of various types of protein elements. This understanding has expanded the possibilities of managing the assembly of both natural proteins and artificial structures in the field of protein engineering, materials science, etc. Due to the biocompatibility of molecular recognition properties and availability for production, biomolecular nanostructures are attractive for use in various fields of biomedicine, biotechnology, and bioengineering. Artificial peptides, such as natural ones, can be targeted for self-assembly to perform a specific function. For example, some of the earliest artificially created peptides in tissue engineering demonstrated that self-organizing peptides are capable of supporting cell attachment and proliferation [1,2]. Other studies have shown that the use of peptides can promote the regeneration of axons and restoration of the brain of animals [3], cultivation of stem cells [4], coordination of lanthanide ions [5] and DNA binding [6]. Peptide nanotubes allow numerous chemical modifications and assist in exploiting the specificity of biological systems. For example, they are used to study the ability of very short aromatic peptides to form ordered amyloid fibrils, which have similar biophysical and structural properties and are a hallmark of a diverse group of diseases (Alzheimer’s disease, type 2 diabetes, prion diseases). The spatial structure and forces of interaction of aromatic fragments provide the direction and energy necessary for these ordered structures’ formation [7]. Therefore, many works studies have been conducted to study the three-dimensional structure of proteins in the context of determining the structural and functional features of regular and irregular protein secondary structures.

Chirality occupies a valuable space in studies of artificial structures, as it is used as a control characteristic of stratification in hierarchies of biomacromolecule structures and, as a consequence, their functional features [8,9]. During the formation of complex protein structures, a chirality sign alternation was identified, from left-handed (L) to right-handed (D), and during the transition between hierarchical levels. However, this pattern requires confirmation in the form of a qualitative and quantitative assessment of chirality for various protein structural levels.

One of the main objectives of protein engineering is to improve protein stability, and this task is associated with the chirality of protein structures. Syndiotactic chains have an enormous ensemble of available conformations; therefore, L- and D-amino acids are often used in bioengineering [10]. Heterochirality is not a characteristic feature of most biological systems. Homochiral amino acid chains, which have significantly fewer possible conformations than heterochiral ones, promote the formation of regular secondary structures since protein isotacticity reduces the entropic component of folding and, accordingly, increases the stability of protein structures [11]. Amino acids of different chirality have different effects on the self-assembly of proteins, and the substitution of enantiomers can alter the kinetics, morphology, and the mechanical properties of self-assembly of the peptides [12,13,14,15,16,17,18,19,20,21]. Thus, a substitution for a D-amino acid is able to disrupt the structure of the helix or β-sheet and destabilize the process of peptide self-assembly [22,23,24,25].

In [26,27], a new self-assembly mode based on the use of helical peptides with a chiral center, where chirality determines the self-assembly of helical structures, is proposed.

A study of the effect of the chirality of amino acids on the structures of diphenylalanine (FF) and its derivatives showed that switching the chirality of one Phe in FF derivatives changed the morphology of their self-assembly but retained the ability to self-assemble into nanotubes, and heterochirality made nanotubes more stable [13,28,29]. At the same time, it was shown that the hydrogel formed by the racemic ferrocene-diphenylalanine mixture was mechanically weaker than the enantiopure hydrogels [30]. Thus, there are differences in self-assembly between racemic mixtures and pure enantiomers. The introduction of D-amino acids into self-organizing L-peptides is widely used to increase the enzymatic stability of structures and affect their biological functions [19].

The left-handed helix of polyproline II (PPII) belongs to the trans-isomers (steric more favorable) of peptide bonds, and the more compact right-handed helix of polyproline I (PPI) belongs to the cis isomers. PPII helices are involved in signal transduction and in the assembly of the protein complex, transcription, protein self-assembly and elasticity, the regulation of many intracellular signaling complexes, and they play a significant structural role in amyloidogenic proteins [31,32].

The formation of sign-alternating chiral structures of different scales can also be observed in cholesteric liquid crystals formed by chiral molecules. In each layer, the molecules are predominantly oriented along the director, and upon passing to the neighbouring layer, the director rotates around the cholesteric axis (rotation is associated with stereospecific molecular restrictions). A helix is formed that is opposite in sign to the chirality of the molecules. “Left” cholesterol defines the dextrorotation of the director [33]. In the cholesteric phase of DNA, a change in the sign of chirality is also observed during the transition to the next level of organization [34]. The cholesteric phase comprises a standard organized parallel layers of DNA molecules. Each layer is rotated relative to the previous layer by a small angle. Right-handed DNA forms layers, which in turn form a left-handed helix.

The combination of flexibility and rigidity within one protein molecule is possibly associated with the aperiodicity of the protein structure crystals [35]. Orientational symmetry is broken in the aperiodic arrangement of secondary structural elements, and the folded structures are nematic droplets. At certain values of the introduced nematic order parameter P_2_, the arrangement of structural elements can withstand mechanical forces. This approach is found to be valid when considering the relationship between the three-dimensional organization and the nematic order of protein allostery.

In this article, we discuss methods for determining the chirality sign of regular (helical) and irregular (turns and loops) protein secondary structures, as well as the possibility of their application for helix-like peptide nanotubes based on amino acids.

## 2. Models and Computational Methods

### 2.1. Objects of Study

#### 2.1.1. Protein Secondary Structures

A particular manifestation of chirality is the helicity of structures. The helix boundaries are determined by a set of amino acids, the sequence of which is encoded in the DNA [8,36]. In this article, three types of regular secondary structures are considered–α, $3_{10}$, and π-helices.

α-helices in natural proteins are more stable and resistant to mutations than β-sheets [37]. The most common protein-regular secondary structure is the right-handed α-helix [38].

The third most common structures after α-helices and β-sheets in globular proteins are $3_{10}$-helices [39]. These short helices are located at the sites of turns of α-helices or their ends [40]. The $3_{10}$-helices have three residues per turn and are less-stable structures than α-helices (possibly due to a slightly different structure of hydrogen bonds) [41]. Similar to α-helices, $3_{10}$-helices are mainly observed in the right-handed conformation.

π-helices are formed as a result of the exclusion or addition of one amino acid residue in the α-helix [42]; they are found in 15% of protein structures [42] and, as a rule, are located near the functional sites of proteins [42]. Thus, 32% of 6-residue π-helices are involved in ligand binding or constitute an active site, and 77% have conserved residues among homologous proteins [43]. The overwhelming majority of natural π-helices consist of seven residues and at least two consecutive π-type H-bonds [44].

Irregular secondary protein structures (turns and loops) act as a link between regular secondary structures [45] and are important elements of molecular recognition in protein folding [46]. The formation of irregular regions is largely determined by the primary amino acid sequence of the polypeptide chain [47]. Turns and coils account for approximately 30% [48] to 50% of the total secondary structure of globular proteins [49]. These structures are often present in the active sites of proteins, facilitating specific interactions between molecules [50] and, as a rule, are located on the surface of a globular protein.

Turns are sufficiently stable structures, since these isolated elements should actively promote the folding and maintenance of the globular form of the protein [51]. Depending on the number of residues separating the pair connected via the hydrogen bond, the turns are divided into δ-, γ-, β-, α-, and π-turns, consisting of 2 to 6 amino acid residues, respectively [52]. In this article, we consider such irregular secondary structures as β- and α-turns, and Ω-loops.

For β- and α-turns, the distance between the first and last α-carbons of the turn is less than 10 Å, and it has no hydrogen bond [52].

A β-turn (of 4 residues) is the most frequent type of turn [52], accounting for 63% of the residues in loops between regular secondary structures and around 25–30% of all protein residues [53]. Even for relatively small peptides, it is believed that the β-turn conformation is bioactive (the rate of β-turns formation is ten times lower than the rate of α-helices formation) [54].

The α-turn is not part of the α-helix. Turns that are not hydrogen bonded contain more hydrophobic residues at *i* and *i +* 4 positions and can provide (and stabilize) hydrophobic interactions between turns. The most common structural motif with an α-turn is a β-hairpin. According to the hypothesis, one of the mechanisms of α-turn initiation is the development of an initial β-turn into an α-turn [55]. The β-turn is important for understanding protein folding mechanisms.

Ω-loops consist of 6–16 amino acid residues, where the lower limit of length serves to exclude reverse turns. As a rule, they are located on the surface of globular proteins, connect membrane α-helices on the cytoplasmic or extracellular surface, and are often involved in recognition processes [56]. On average, a protein molecule contains around four Ω-loops, the distance between the ends is less than the α-α carbon separation in a loop, the twisting angles of the main chain are not repeated, and there are fewer hydrogen bonds of the main chain [57]. The hydrogen bond in the main chain of the loop is irregular, which favors the packing of side chains within long loops [51]. Therefore, depending on the three-dimensional shape, Ω-loops exhibit different degrees of flexibility during protein folding [58] and affect the function of protein structures [59]. Furthermore, Ω-loops can be used in bioengineering since their replacement or elimination affects the stability and enzymatic activity of the protein.

It is clear that the identification of irregular structures in proteins is necessary for understanding their structure and functions since they connect secondary structural elements, change the direction of the polypeptide chain, and often contain residues of active sites.

#### 2.1.2. Peptide Nanotubes

One example of the self-assembly of complex biomolecular structures is the formation of a helical nanotube-type structure based on a phenylalanine amino acid (F or Phe) [60]. It is known that, based on such a phenylalanine amino acid, dipeptides, diphenylalanine (FF or (Phe)_2_), are also formed, which are then easily assembled into peptide nanotubes [61,62,63]. Diphenylalanine dipeptide and peptide nanotubes (PNT), based on this process (FF PNT), are currently well studied, as they are of considerable interest due to their special structural and physical properties, which are important in various applications. However, single phenylalanine molecules can also form nanotubes and nanofibrils [64,65]. In [60], the modeling and assembly of a set of (Phe)_48_ molecules into a helix-like tubular structure of a phenylalanine peptide nanotube (F48 PNT) using the molecular dynamics method (MD manipulator) was considered. Data on the formation of nanotubes of the “right” (D-F48 PNT) chirality from the initial L-F peptides and nanotubes of the “left” (L-F48 PNT) chirality from the D-F peptides were obtained.

In this work, using the approach described in [66], based on the values of the dipole moments of individual peptides, we calculated the sign and magnitude of chirality for such a phenylalanine spiral nanotube [60].

### 2.2. Methods for Evaluating the Chirality of Regular Helical and Irregular Protein Secondary Structures

According to M. Petitjean, the measure of chirality should be of a continuous characteristic and should be determined for a space of any dimension, and the chirality index should not depend on the method of selecting the mirror image [67]. At present, there are a significant number of works in which various methods for evaluating the chirality of helical structures are proposed. The main methods are as follows: a “connectivity index” for alkanes depending on physical parameters [68,69,70], binary code for benzenoids in 2D space [71], the deviation of a chiral set from a reference achiral set [72], chirality as overlapping the initial set with its mirror image [73], a measure of continuous symmetry based on determining the distance from a distorted molecular shape to a selected symmetry element [74,75], pseudoscalar measures of electronic chirality for molecular systems using the rotational polarizability of molecules [76], calculating the “degree of chirality” based on the overlap and an infinite hierarchy of pseudoscalar parameters for spiral ribbons, using a two-dimensional plane [77]. The described methods are often highly specialized and, as a rule, provide an estimate of symmetry, not chirality, and are fairly difficult to calculate, even if they are not based on experiments. The folding of peptide chains by rotating the planes of peptide bonds, relative to their bonds with α-carbon atoms, is described by dihedral angles, the values of which provide the basis for assessing the chirality of proteins in the Ramachandran method, which allows for the observation of the predominant conformation of elements of the secondary structure of biomolecules [78]. Since its inception, this method has become the main method for characterizing proteins. In [79], the structural RP-analysis of proteins was extended from a two-dimensional map to a three-dimensional one, and a quantitative degree of chirality was added as a continuous measure of chirality (CCM), covering all bond angles and bond lengths of the amino acid residue at each point of the map. The addition of chirality to the Ramachandran plots made it possible to emphasize the sensitivity of the protein structure to minor conformational changes. It has been shown that points with higher values of chirality are special transition points in a protein, such as bends of the α-helix, twisting of β-chains [80]. The authors of [81] presented a method based on the consideration of a peptide framework as a helix with axial (d) and angular (θ) displacements, which were obtained based on the co-ordinates of the peptide framework and dihedral angles. The method is used to characterize each region of the Ramachandran plot for both cis (ω = 0°) and trans (ω = 180°) peptides.

There are several main approaches used in recognizing and modeling turns and loops. In [82], the parameters of loops and turns that are similar in the length and geometry of their endpoints are used. The mechanisms for searching for structural motifs are described in [83]: the extension of the DeepView/Swiss-PdbViewer algorithms allows for the determination of structural motifs in a large databases of protein structures. The knob-socket model serves to determine the role of coils and turns within a tertiary structure. In the works of various authors for β-turns, the dihedral angles φ, ψ are calculated on the Ramachandran plots [47,84,85,86,87,88,89]. Several statistical and computational methods for determining the structure of γ-turns are described. In particular, in [90], a method for recognizing γ-turns using neural networks was proposed. The determination of the structural characteristics of the loops seems to be especially difficult since their residues do not correspond to the pattern of dihedral angles or hydrogen bonds. The average prediction accuracy is primarily limited by the accuracy of the energy function, not by the degree of conformational sampling [91]. Attempts to determine rotations by including the interaction with the participation of atoms of the side chains in the analysis were made [92,93].

**Figure 1:**
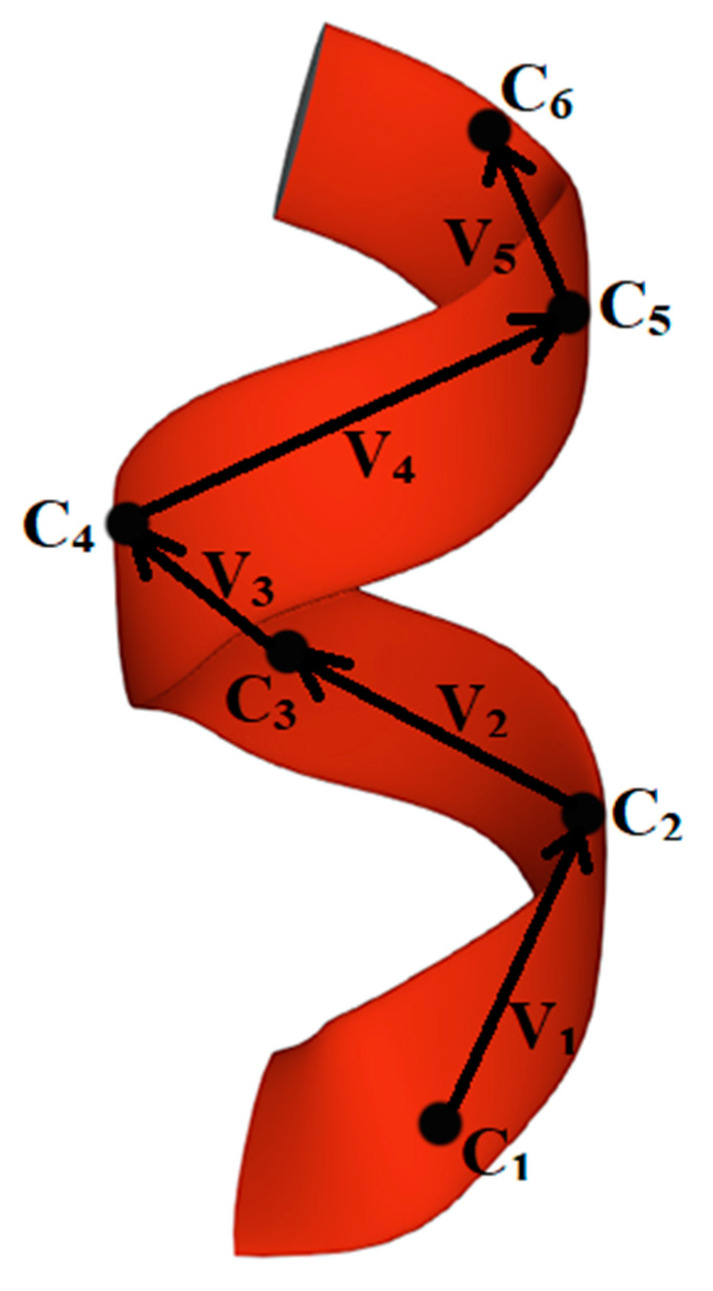
Graphic construction of vectors $v_{i}$ for calculating the mixed vector product of helical protein structure (1L63 [96], α-helix, residues 45–50). $C_{i}$–atoms of α-carbons, reference points in helix.

We considered the estimated chirality of the helices in [94,95]. In this paper, we describe in more detail the mathematical aspects of the solution. According to the mentioned method for the assessment of the chirality sign of helical structures, the mutual arrangement of α-carbons-reference points in the helices provides a sufficient condition [66,94,95]. The chirality sign of secondary helical structures can be assessed by the sum of mixed products for triples of vectors ($v_{i}$) built between successive reference points Cα as follows:

(1) $$\chi_{total}=\sum_{i=1}^{n-3}\left({[v}_{i},v_{i+1}],\;v_{i+2}\right),$$

where the original vectors are calculated by taking into account the Cα coordinates presented in the PDB (Figure 1)

(2) $$\left(\left[v_{1},v_{2}\right],v_{3}\right)=\left(y_{1}z_{2}-y_{2}z_{1}\right)x_{3}+\left(z_{1}x_{2}-z_{2}x_{1}\right)y_{3}+\left(x_{1}y_{2}-x_{2}y_{1}\right)z_{3}.$$

In [94], we proposed a model for the normalized value of the chirality of protein helical structures as follows:

(3) $$\chi_{norm}=\sum_{i=1}^{n-3}\frac{\left({[v}_{i},v_{i+1}],{\;v}_{i+2}\right)}{C_{i}},$$

where the normalization factor is calculated as $C_{i}={\left(\frac{1}{3}\overset{2}{\underset{j=0}{\sum }}\left|v_{i+j}\right|\right)}^{k}$.

The chirality normalization was calculated based on considering the chirality value of the helix as a physical object, which should tend to a certain value with an increase in the number of reference points (that is, with an increase in the density of points). Therefore, each mixed product is normalized to the power *k* of the average length of the vectors.

**Figure 2:**
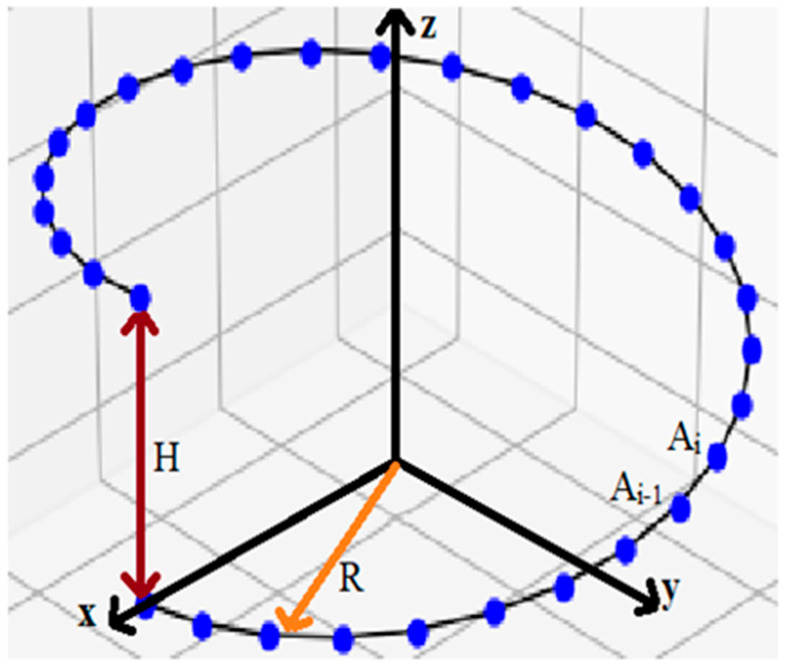
Normalization of chirality. The blue dots are reference points for this structure.

To find the value of *k*, we establish the behavior of the chirality characteristic χ when the length of the vectors connecting the reference points changes. To execute this, consider a helix of radius *R* and height *H*. Let there be *N* reference points on this turn. We then performed all calculations in cylindrical coordinates. The origin of the coordinates coincides with the middle of the turn (Figure 2).

Then, the coordinates of the *i*-th point of the turn (denote it $A_{i\;}$):

(4) $$A_{i\;}:\left(R\cos\varphi_{i};R\sin\varphi_{i};ih\right),\;where\;i=0\ldots N,\;h=\frac{H}{N},\varphi_{i}=i\varphi \;\left(\varphi =\frac{2\pi }{N}\right).$$

In accordance with this, the coordinates of the $\vec{r_{i}\;}$ vector connecting the points $A_{i-1}$ and $A_{i}$ have the following form:

(5) $$\vec{r_{i}\;}:\;\left\{R\cos\varphi_{i}-R\cos\varphi_{i-1};R\sin\varphi_{i}-R\sin\varphi_{i-1};h\right\}.$$

Consider separately the *x* and *y* coordinates of the $\vec{r_{i}\;}$ vector:

(6) $$x_{i}=R\left(\cos\varphi_{i}-\cos\varphi_{i-1}\right)=-2\sin[\frac{1}{2}\left(\varphi_{i}+\varphi_{i-1}\right)]\sin[\frac{1}{2}\left(\varphi_{i}-\varphi_{i-1}\right)]=-2\sin[\left(2i-1\right)\varphi ]\sin\frac{\varphi }{2},$$

(7) $$y_{i}=R\left(\sin\varphi_{i}-\sin\varphi_{i-1}\right)=2\sin[\frac{1}{2}\left(\varphi_{i}-\varphi_{i-1}\right)]\cos[\frac{1}{2}\left(\varphi_{i}+\varphi_{i-1}\right)]=2\sin\varphi \cos\left[\left(2i-1\right)\varphi \right].\;$$

Using the obtained values for the coordinates, we calculate the modulus (length) of the $\vec{r_{i}\;}$ vector:

(8) $$\begin{array}{cc} \left|\vec{r_{i}\;}\right| & =\sqrt[{2}]{x_{i}{}^{2}+y_{i}{}^{2}+z_{i}{}^{2}}=\sqrt[{2}]{h^{2}+R^{2}{\left(\cos\varphi_{i}-\cos\varphi_{i-1}\right)}^{2}+R^{2}{\left(\sin\varphi_{i}-\sin\varphi_{i-1}\right)}^{2}}\\ & =\sqrt{h^{2}+4{\left(\sin\frac{\varphi }{2}\right)}^{2}R^{2}\left(\sin{\left[\frac{\left(2i-1\right)\varphi }{2}\right]}^{2}+\cos{\left[\frac{\left(2i-1\right)\varphi }{2}\right]}^{2}\right)}=\sqrt{h^{2}+4{\left(\sin\frac{\varphi }{2}\right)}^{2}R^{2}.} \end{array}$$

The chirality characteristic, as already mentioned, is calculated using the following formula:

(9) $$\chi =\overset{N-3}{\underset{i=1}{\sum }}\left(\left[\vec{r_{i}\;},\vec{r_{i+1}\;}\right]\vec{,r_{i+2}\;}\right).$$

Consider the vector product in (6) and take into account that the coordinates of the vectors are calculated by Formulas (3) and (4). Then

(10) $$\begin{array}{cc} \left[\vec{r_{i}\;},\vec{r_{i+1}\;}\right] & =\left|\begin{array}{ccc} i & j & k\\ x_{i} & y_{i} & z_{i}\\ x_{i+1} & y_{i+1} & z_{i+1} \end{array}\right|\\ & =\vec{i}hR\left(2\sin\frac{\varphi }{2}\cos\frac{2i-1}{2}\varphi -2\sin\frac{\varphi }{2}\cos\frac{2i+1}{2}\varphi \right)\\ & +\vec{j}hR\left(2\sin\frac{\varphi }{2}\sin\frac{2i-1}{2}\varphi -2\sin\frac{\varphi }{2}\sin\frac{2i+1}{2}\varphi \right)\\ & +\vec{k}4R^{2}\left(\sin\frac{\varphi }{2}\sin\frac{2i+1}{2}\varphi \sin\frac{\varphi }{2}\cos\frac{2i-1}{2}\varphi -\sin\frac{\varphi }{2}\cos\frac{2i+1}{2}\varphi \sin\frac{\varphi }{2}\sin\frac{2i-1}{2}\varphi \right). \end{array}$$

Substituting Formula (7) into Equation (6), we obtain:

(11) $$\begin{array}{cc} \left([\vec{r_{i}\;},\vec{r_{i+1}\;}]\vec{,r_{i+2}\;}\right) & =4hR^{2}{\left(\sin\frac{\varphi }{2}\right)}^{2}[-\left(\cos\frac{2i-1}{2}\varphi -\cos\frac{2i+1}{2}\varphi \right)\sin\frac{2i+3}{2}\varphi \\ & +\left(\sin\frac{2i-1}{2}\varphi -\sin\frac{2i+1}{2}\varphi \right)\cos\;\frac{2i+3}{2}\varphi +(\sin\frac{2i+1}{2}\varphi \cos\frac{2i-1}{2}\varphi \\ & -\cos\frac{2i+1}{2}\varphi \sin\frac{2i-1}{2}\varphi )].\; \end{array}$$

Let us consider separately several terms in (11), taking into account trigonometric transformations:

(12) $$\cos\frac{2i-1}{2}\varphi \sin\frac{2i+3}{2}\varphi =\frac{1}{2}\left(\sin\left(2i+1\right)\varphi +\sin2\varphi \right),$$

(13) $$\cos\frac{2i+1}{2}\varphi \sin\frac{2i+3}{2}\varphi =\frac{1}{2}\left(\sin\left(2i+1\right)\varphi +\sin2\varphi \right),$$

(14) $$\sin\frac{2i-1}{2}\varphi \cos\frac{2i+3}{2}\varphi =\frac{1}{2}\left(\sin\left(2i+1\right)\varphi -\sin2\varphi \right),\;$$

(15) $$\sin\frac{2i+1}{2}\varphi \cos\frac{2i+3}{2}\varphi =\frac{1}{2}\left(\sin\left(2i+2\right)\varphi -\sin\varphi \right),$$

(16) $$\sin\frac{2i+1}{2}\varphi \cos\frac{2i-1}{2}\varphi =\frac{1}{2}\left(\sin2i\varphi +\sin\varphi \right),$$

(17) $$\sin\frac{2i-1}{2}\varphi \cos\frac{2i+1}{2}\varphi =\frac{1}{2}\left(\sin2i\varphi -\sin\varphi \right).$$

By opening the brackets and substituting Formulas (12)–(17) into (11), we obtain an expression for the mixed product:

(18) $$\begin{array}{cc} \left(\left[\vec{r_{i}\;},\vec{r_{i+1}\;}\right],\vec{r_{i+2}\;}\right) & =2hR^{2}{\left(\sin\frac{\varphi }{2}\right)}^{2}[\sin\left(2i+2\right)\varphi +\sin\varphi -\sin\left(2i-1\right)\varphi \\ & -\sin2\varphi +\sin\left(2i+1\right)\varphi -\sin2\varphi -\sin\left(2i+2\right)\varphi +\sin\varphi +\sin2i\varphi +\sin\varphi -\sin2i\varphi \\ & +\sin\varphi ]=2hR^{2}{\left(\sin\frac{\varphi }{2}\right)}^{2}\left[4\sin\varphi -2\sin2\varphi \right].\; \end{array}$$

The final expression for characterizing chirality is obtained by a summation of all the mixed products:

(19) $$\chi =\overset{N-3}{\underset{i=1}{\sum }}\left(\left[\vec{r_{i}\;},\vec{r_{i+1}\;}\right],\vec{r_{i+2}\;}\right)=16\left(N-3\right)HR^{2}{\left(\sin\frac{\varphi }{2}\right)}^{4}\sin\varphi .$$

Consider the behavior of *χ* (19) and $\left|\vec{r}\right|$ (8) as the number of reference points tends to infinity:

(20) $$\underset{N\to \infty }{\lim}\chi =\underset{N\to \infty }{\lim}16\left(N-3\right)HR^{2}{\left(\sin\frac{\pi }{N}\right)}^{4}\sin\frac{2\pi }{N}=\underset{N\to \infty }{\lim}\frac{32\left(N-3\right)HR^{2}\pi^{5}}{N^{5}}\sim \frac{1}{N^{5}},$$

(21) $$\underset{N\to \infty }{\lim}\left|\vec{r\;}\right|=\underset{N\to \infty }{\lim}\sqrt{{\frac{H}{N^{2}}}^{2}+4{\left(\sin\frac{\pi }{N}\right)}^{2}R^{2}}=\underset{N\to \infty }{\lim}\frac{1}{N}\sqrt{H^{2}+4\pi^{2}R^{2}}\sim \frac{1}{N}.$$

Thus, as $N\to \infty \;\chi \sim {\left|\vec{r\;}\right|}^{5}$, therefore,

(22) $$\chi_{norm}=\frac{\chi }{{\left|\vec{r\;}\right|}^{5}}.$$

Thus, to preserve the finite nonzero chirality with an unlimited increase in the number of points, one should take *k* = 5.

**Figure 3:**
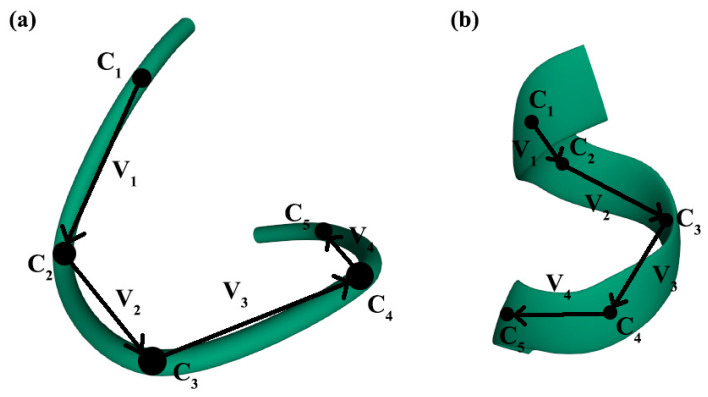
Graphic construction of $v_{i}$ vectors for calculating the mixed vector product, α-turns of the 2FOX protein [97]: (**a**) α-turn, type g4 [98]; (**b**) α-turn, type g5 [98] (the studied types of α-turns are discussed in the Results section). $C_{i}$ –atoms of α-carbons, reference points in α-turns.

**Figure 4:**
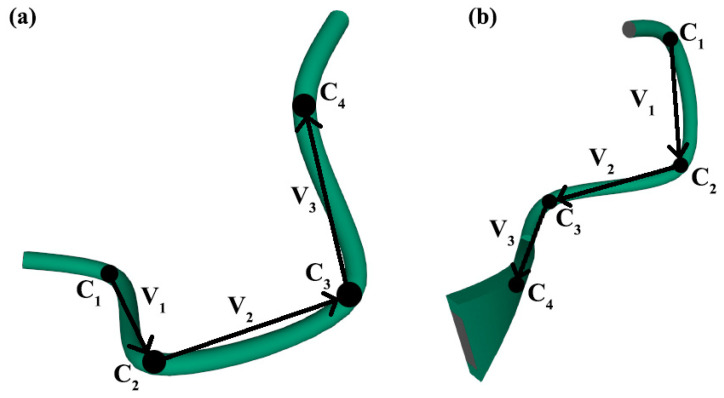
Graphic construction of $v_{i}$ vectors for calculating the mixed vector product, β-turns of the 1A4G protein [99]: (**a**) β-turn, type AG [89]; (**b**) β-turn, type Dd [89] (the studied types of β-turns are discussed in the Results section). $C_{i}$ –atoms of α-carbons, reference points in β-turns.

**Figure 5:**
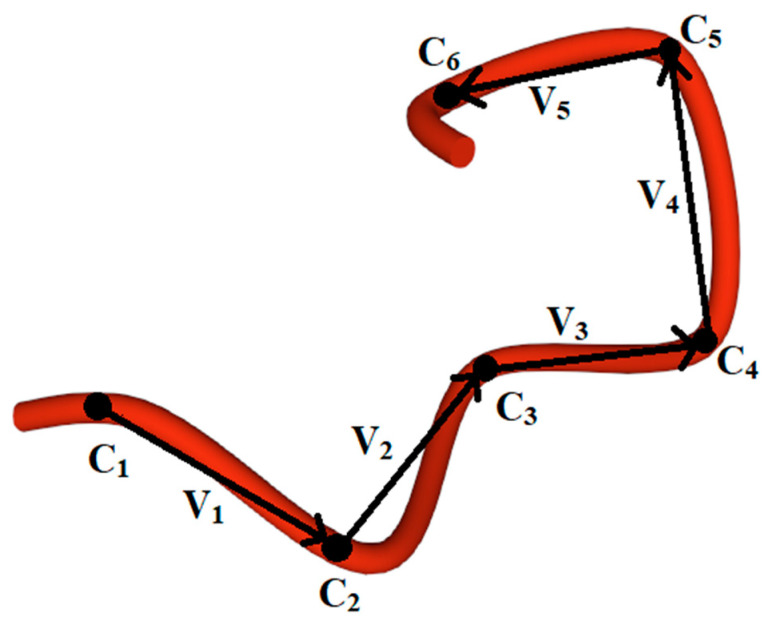
Graphic construction of $v_{i}$ vectors for calculating the mixed vector product, Ω-loop of the 2ACT protein [100] (residues 8–13). $C_{i}$ –atoms of α-carbons, reference points in the Ω-loop.

The assessment of the chirality of irregular protein secondary structures is similar to that of regular structures. In this article, the chirality of irregular protein secondary structures was assessed using the example of β-, α-turns and Ω-loops (Figure 3, Figure 4 and Figure 5). A sufficient condition is provided by the relative position of the α-carbon reference points in turns and loops.

The results of calculating the chirality of regular (helical) and irregular (turns and loops) protein secondary structures are presented in the Results section.

### 2.3. Method for Calculating the Chirality of Phenylalanine (F) Helical Peptide Nanotubes (PNT) from Successive Dipole Moments of Their Constituent Phenylalanine Molecules

Recently, in [60], a new approach was proposed for modeling various molecular nanostructures, determining the implementation of the molecular dynamics simulation (MDS) run trajectory, and forming the final structure using the so-called molecular dynamics manipulator (MDM). This approach is a type of MDS, developed based on the PUMA-CUDA software package [101,102], using the physics of the PUMA software package [103,104]. Using this tool allows for the exploration of the formation process of helical structures from a linear sequence of any amino acids. It was used in [60] to assemble nanotubes from linear phenylalanine chains of different chirality (L-F and D-F) by including additional force effects in the molecular dynamics simulation program for these structures.

In this work, using the obtained helical structures of phenylalanine nanotubes of different chirality [60], we calculate the magnitude and sign of their chirality using a method similar to that developed in [94,95] and applied in [66] to diphenylalanine helical structures, based on the procedure of the mixed product of three consecutive vectors in a coil of a spiral structure. To achieve this, we select one coil of the helix from each nanotube of different chirality and apply this calculation method to them based on the mixed product of vectors of dipole moments from a number of successive phenylalanine molecules that form this coil of the helix of a phenylalanine peptide nanotube (PNT), taking into account its chirality.

**Figure 6:**
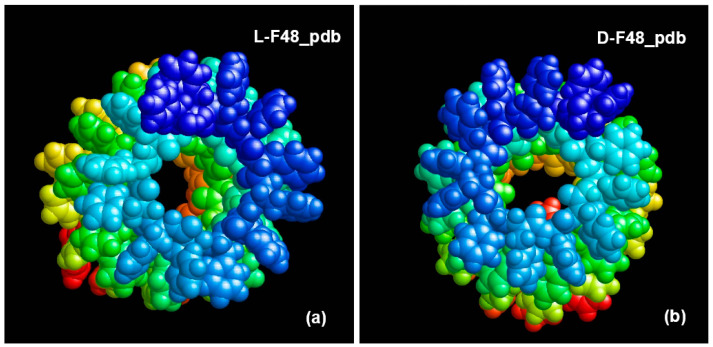
The obtained results of MDS and self-assembly of phenylalanine helical-like nanostructures: (**a**) L-F48_pdb; (**b**) D-F48_pdb (images were obtained from *.pdb files using the RasMol program http://www.openrasmol.org/ (accessed on 30 September 2021)).

Helical-like PNT structures, based on phenylalanine of different chirality (L and D), were obtained as a result of MD simulation (MDS) and their assembly [60], and consist of 48 F molecules and 4 coils in such structures of nanotubes of each chirality L-F48 and D-F48 (with coordinates of all atoms in standard *.pdb format) (Figure 6, Video S1).

**Figure 7:**
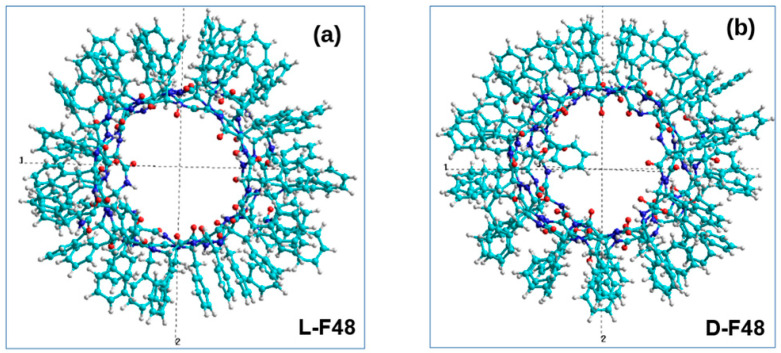
The obtained results of MDS and self-assembly of phenylalanine helical-like PNT nanostructures, transferred to HyperChem workspace (in Z-projection): (**a**) F48L PNT; (**b**) F48D-PNT.

We transferred dipole moments to the HyperChem [105] workspace (in *.hin format with Cartesian x, y, z coordinates for all atoms) for analysis (Figure 7). 

**Figure 8:**
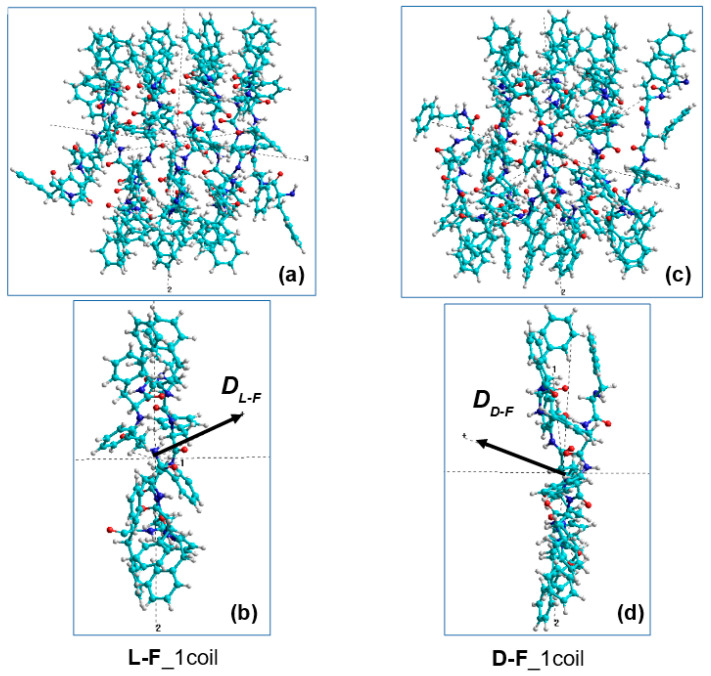
Selection of the one coil from the four in PNT helix-like structures (in X-projection): (**a**, **b**)–for the L-F PNT; (**c**, **d**)–for the D-F PNT.

We selected an individual coil at sequentially from each PNT helix consisting of four coils. When calculating the dipole moments of these coils we found that they have opposite directions to the vectors of the total dipole moments **D_L-F_** for each L-F and **D_D-F_** for each D-F coils (Figure 8b,d).

**Figure 9:**
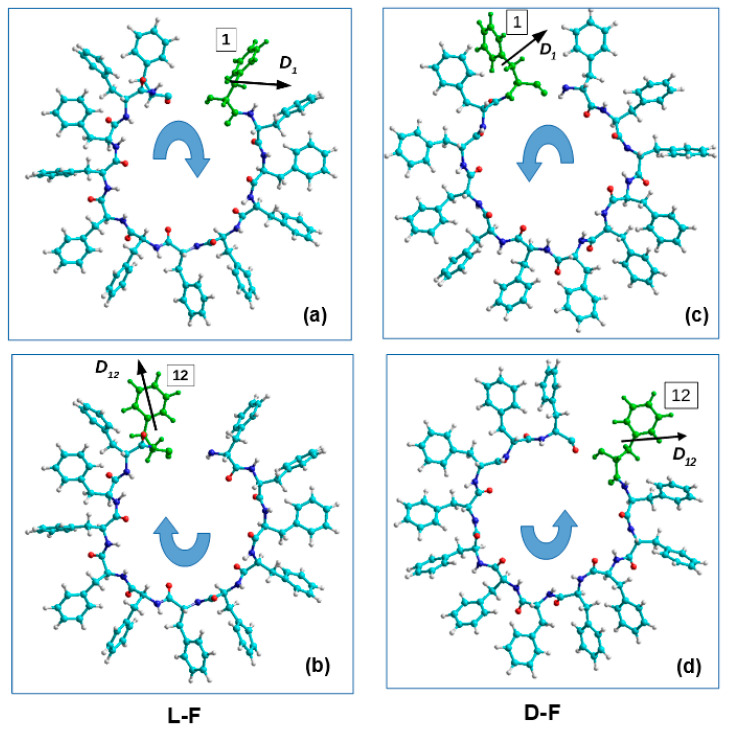
Schematic of the selection procedure of each consequential phenylalanine F molecule (from 1 up 12) from one corresponding coil of helix and the calculation of its dipole moment **D_*i*_** (for i = 1, …, 12) (in Z-projections): (**a**,**b**)–for the L-F PNT; (**c**,**d**)–for the D-F PNT, correspondingly.

We then selected each consequential phenylalanine F molecule from corresponding coil, containing 12 F molecules, and calculated its dipole moment **D_*i*_** using various methods (from HyperChem package [105,106]), including the following: (1) quantum-chemical semi-empirical RM1 RHF [106]; (2) classical molecular mechanical Amber method [105]. This procedure schematically shown in Figure 9.

Similarly to the calculation of diphenylalanine nanotubes [66], we use a similar successive set of F molecules for phenylalanine nanotubes. The origin of **D*_i_*** vectors is obtained relative to the center of mass of the corresponding molecules. The absolute value of each dipole moment **D***_i_* is

(23) $$D_{i}=\left|D_{i}\right|=\sqrt{D_{x,i}^{2}+D_{y,i}^{2}+D_{z,i}^{2}},\;$$

where *D_x,i_, D_y,i_,* and *D_z,i_* are the components of the *i*-th vector **D*_i_*** in the Cartesian coordinates. Similar to Equation (1) [94,95], the sum of the scalar triple products of the dipole moments related to the PNT’s chirality can be written as:

(24) $$c_{total}=\overset{n-2}{\underset{i=1}{\sum }}\left(\left[D_{i},D_{i+1}\right],D_{i+2}\right),\;$$

It is necessary to note that the summation here has taken over *i* in the range from 1 to n-2, whereas in Equation (1), the *i* range is from 1 to n-3. Now *n* = 12. This is because in supramolecular helixes *i* numerates the individual molecules instead of the Cα atoms in proteins. The *c_total_* can be normalized over the average value of the total dipole momentum of the PNT’s coil, $D_{av}=\frac{1}{12}\overset{12}{\underset{i=1}{\sum }}D_{i}$, to obtain a universal measure of the chirality:

(25) $$c_{norm}=\frac{c_{total}}{D_{av}^{3}}.\;$$

Individual dipole moments of F molecules in one coil of helical PNTs were calculated using the semi-empirical quantum-mechanical method PM3 in the restricted Hartree–Fock approximation (RHF) and molecular force field method Amber from the HyperChem package [105,106]. The results for L-F and D-F are shown below in the Results section. A schematic representation of the spatial arrangement of F of the individual dipole moments $D_{i}$ in two PNT coils is presented in Figure 9a–d for L-F and for D-F PNT. The obtained results of calculating the magnitude and sign of chirality by Formulas (23)–(25) for each case (L and D) are presented in the Results section.

## 3. Results

### 3.1. Helical Protein Secondary Structures

**Figure 10:**
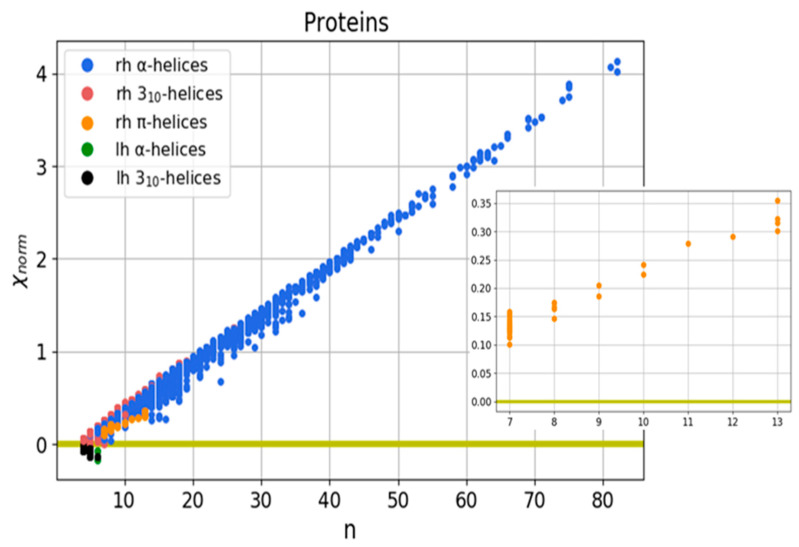
Normalized chirality map of protein helical structures. The horizontal axis is the length of the secondary structure in amino acid residues, the vertical axis is the normalized chirality value. The inset shows a map of π-helices; rh–right-handed structures, lh–left-handed structures.

Using the developed method, files from PDB with the data of 983 proteins of various classes were considered. The chirality of helical structures was calculated, and data for α- and $3_{10}$-helices (oxidoreductase, transferase, hydrolase, lyase, isomerase, ligase, translocase, chaperones, viruses, structural proteins, endo- and exocytosis proteins, electron transport-protein data of chirality) are presented in [95], as well as for π-helices (all π-helices are taken from [44]). In accordance with Formula (3), a map of normalized chirality for the considered helical protein structures is presented (Figure 10).

The results obtained showed that for all the considered regular protein structures (helices), the developed measure of chirality ($\chi_{norm}$) linearly depends on the number of atoms in the helix. Secondly, the results obtained are consistent with the literature data on the predominant conformation of right-handed helical structures.

The calculated data for α and $3_{10}$-helices were presented previously in our work [95]. The calculated parameters of π-helices are presented in Table A1.

### 3.2. Irregular Protein Secondary Structures

We estimated the chirality of α-turns (Figure 3) on the basis of the data presented in [98], where all α-turns are classified depending on the value of the torsion angles (φ, ψ) for various amino acid residues that comprise the turns. Based on this, the authors distinguish 9 types of α-turns, including two types of F1 and F2, called families, as well as seven less common types g1, g2, g3, g4, g5, g6, g7, termed groups. In addition, the two turns identified were not included in any of the groups. We examined 78 α-turns identified by the authors of [98], calculated the chirality for each turn using our described method, and obtained the mean values and standard deviations of chirality for each type. It follows that most of these types have certain chirality values characteristic of each type of α-turn. The calculation results are presented in Table 1. The classes for α-turns are presented in Table A2.

For β-turns (Figure 4), we performed similar calculations using the database presented in [89]. Article [89] presents a new classification of β-turns based on an algorithm for their identification and recognition, implemented in the form of a computer program. The authors divided all β-turns into 18 types, in addition to those that were not included in any of the newly formed groups, based on the following criteria: the distance between the first and last residues of turn, the values of torsion angles (φ, ψ) for the second and third amino acid residues, and the conformation of these residues relative to peptide bonds (cis/trans). Using the computer program of the authors of [89], we distinguished 850 uniquely determined β-turns from 20 proteins taken from the PDB [107] and calculated the mean values and standard deviations of chirality for each type (Table 2).

**Figure 11:**
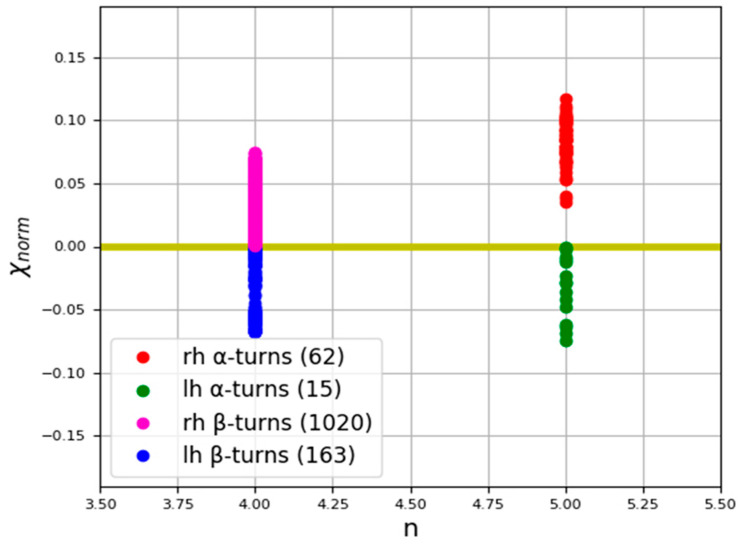
Normalized chirality map of α- and β-turns (mean values of various types presented in Table 1 and Table 2). The horizontal axis is the length of the secondary structure in amino acid residues, the vertical axis is the normalized chirality value; rh–right-handed structures, lh–left-handed structures.

The calculation results showed that, for most types of β-turns, there are certain ranges of chirality values, which also confirms the correctness of such a classification of these structures s found in [89]. Based on the calculations performed (Table 1, Table 2 and Table A2), a chirality map was obtained for the considered α- and β-turns (Figure 11).

An analysis of the normalized chirality map of α- and β-turns (Figure 11) indicates that, for all the considered proteins, the measure of chirality ($\chi_{norm}$) of turns linearly depends on the number of atoms in these structures (see data in Table 1 and Table 2). However, since all considered β-turns consist of 4 amino acid residues, and α-turns consist of 5 residues, they are located parallel to the Y-axis on the map.

**Figure 12:**
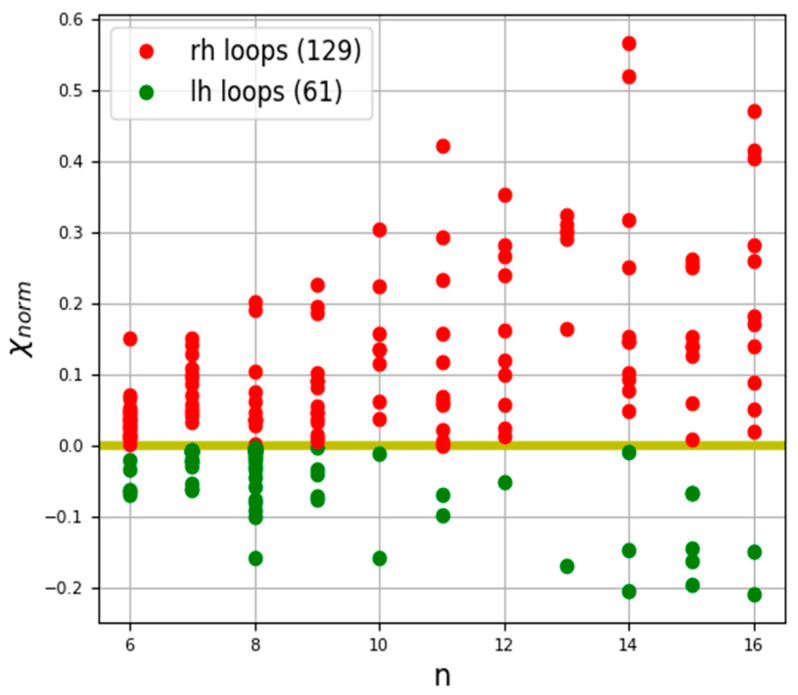
Normalized chirality map of Ω-loops. The horizontal axis is the length of the secondary structure in amino acid residues, the vertical axis is the normalized chirality value; rh–right-handed structures, lh–left-handed structures.

To calculate the chirality of the Ω-loops (Figure 5), the data in [57] were utilized. The chirality calculation data for 190 Ω-loops are presented on the chirality map (Figure 12) and in Table A3.

The spatial orientation of the loops clearly affects the quantitative values of the chirality of these structures. Let us consider a stepwise change in chirality in the process of calculating the total chirality of the loop structure using the example of a loop from the 2ACT protein (Figure 5). Depending on the number of consecutive residues used for calculation, the chirality can be altered either upwards or downwards (Table 3).

### 3.3. Phenylalanine (F) Helix-like Peptide Nanotubes

Following the method for calculating the chirality of phenylalanine (F) helix-like peptide nanotubes (PNT), the results of calculating the dipole moments for the sequence of individual amino acids of phenylalanine F from the turns of spiral nanotubes of different chirality are presented. Nanotubes were obtained by coiling a linear sequence of amino acid F, initially with a different chirality of L-F and D-F (by the molecular dynamics method according to [60]). The obtained results of calculating the magnitude and sign of chirality by Formulas (23)–(25) for each case (L and D) are presented in Table 4, Table 5 and Table 6.

Table 6 shows the magnitude and sign of chirality, calculated using the formulas for the mixed product of dipole moments (23)–(25).

The obtained results (Table 6) reveal a characteristic change in the sign of chirality during transition to a higher level of organization, which is observed in the structures of biomacromolecules [8,9]. The calculated chirality of a spiral nanotube, based on the L-F initial amino acid, was found to have a positive sign-D type, and the chirality of the D-F-based nanotube has a negative sign corresponding to the L chirality type.

Note that the data presented here for the cube of average values for the absolute value of the total dipole moment $D_{av}=\frac{1}{12}\overset{12}{\underset{i=1}{\sum }}D_{i}$ of each of the F amino acids, represent the average volume built on three consecutive vectors of the mixed product of these vectors. For each group of three such vectors, the calculated value of the magnitude of their mixed product changes, corresponding to the volume on these three vectors. hereby normalizing for the average value (*D_av_*)^3^ according to Formula (25), we obtain the relative change in volume, with a slight change around “1”. Moreover, for the left-handed and right-handed triplets of vectors, the corresponding volumes have different signs, which leads to a change in sign in this case.

## 4. Discussion

A chirality analysis was conducted for 26,150 helical structures, namely, 21,702 α-helices, 4360 3_10_-helices, and 88 π-helices (all studied π-helices were taken from [44]). Research has shown that most of the helical structures are right-handed. Among the structures studied, we found 21,689 right-handed α-helices, 4160 right-handed 3_10_-helices, and 88 right-handed π-helices. To study the chirality of π-helices, 84 proteins were analyzed, including 23 oxidoreductases, 22 hydrolases, 8 lyases, 7 transferases, 5 isomerases, 4 binding proteins, 2 toxins, 2 photosynthetic proteins, 2 signal proteins, 1 electron transport protein, 1 luminescent protein, 1 viral protein, 1 protein of endo- and exocytosis, 1 oxygen transport protein, 1 ion transport protein, 1 antibiotic, 1 adhesion protein and 1 iron transport protein (Table A1).

The obtained results show that, for regular helical protein secondary structures (the data for the chirality of α– and 3_10_– helices are presented in [95]), the data for π-helices are in Table A1), the developed measure of chirality (χ_norm_) linearly depends on the number of atoms in the helix (Figure 10). For irregular protein secondary structures (β-turns-Table 2, α-turns-Table A2, Ω-loops-Table A3), a different picture emerges. Since all of the considered β-turns consist of 4 amino acid residues, and α-turns consist of 5 residues, they were found to be located parallel to the Y-axis on the chirality map (Figure 11). The spatial orientation of Ω-loops, in contrast to turns, consists of different numbers of amino acid residues, but unlike regular helices, they are characterized by different spatial orientations. These features affect the quantitative values of the chirality of the loops, whereby, depending on the number of consecutive residues taken for the calculation, the chirality can be altered either upwards to or downwards (Figure 12, Table 3).

Irregular protein secondary structures connect regular protein secondary structures and play a key role in the formation of a protein globule [56]. Given the frequency of occurrence of α-, β-turns, and Ω-loops, this study acts as a useful tool for studying the structure of proteins, both natural and artificial, as well as for protein design and materials science.

The results obtained for calculating the magnitude and sign of chirality, for L- and D-nanotubes based on phenylalanine (Table 4, Table 5 and Table 6), are similar to the data of other works on modeling peptide and dipeptide nanotubes of different chirality and experimental data [61,62,63,108]. These data also fully comply with the regularity of the change in the chirality sign of molecular structures with the complication of their hierarchical level of organization [8,9]. Therefore, this method for calculating the magnitude and sign of chirality by the mixed product method of Sidorova et al. [94,95], using the values of dipole moments in the sequence of individual peptides and dipeptides (or amino acids) as vectors, is found to be suitable, and can be successfully applied for assessments on the magnitude and sign of chirality of complex self-organizing helix-like nanostructures based on amino acids, as well as peptides and dipeptides.

## Figures and Tables

**Table 1:** Mean values of chirality and standard deviations for α-turns of various types [98], calculated using the method for evaluating the chirality of regular helical and irregular protein secondary structures.

Type of α-Turn	Number	Mean Chirality Value	Standard Deviation
F1	46	0.08628	0.01473
F2	8	0.06922	0.013
g1	5	−0.03274	0.00846
g2	2	−0.04165	0.00864
g3	4	0.02838	0.02813
g4	4	−0.06686	0.00568
g5	3	0.10221	0.00191
g6	3	−0.00376	0.00415
g7	2	0.09378	0.02175
Other	2	0.0323	0.05972

**Table 2:** Mean values and standard deviations of chirality for β-turns of various types (according to [89]), calculated using the method for evaluating the chirality of regular helical and irregular protein secondary structures. The proteins with β-turns are hydrolases.

Type of β-Turn	Number	Mean Chirality Value	Standard Deviation
AD	481	0.05041	0.01634
Pd	61	0.02132	0.01371
Pa	36	0.00392	0.02101
ad	60	−0.05916	0.00556
AB1	17	0.00647	0.01820
AZ	16	0.04043	0.01255
AB2	3	0.04561	0.00151
pD	30	−0.01805	0.02312
AG	6	0.06884	0.00086
BcisP	10	0.03700	0.01090
dD	5	0.05961	0.01223
PcisD	10	0.00591	0.01596
dN	6	0.05235	0.00206
Dd	5	−0.06710	0.00107
cisDA	6	0.06506	0.00022
pG	7	0.00086	0.02898
cisDP	3	0.04490	0.02358
other	88	0.02855	0.03472

**Table 3:** Stages of calculating the chirality value of the Ω-loop, 2ACT protein (residues 8–13) [100].

Number of Residues	Number of Vectors	Number of Mixed Products	Addition to the Chirality Value at This Step	Total Chirality Value at This Step
1	--	--	0	0
2	1	--	0	0
3	2	--	0	0
4	3	1	0.0554	0.0554
5	4	2	−0.0631	−0.0077
6	5	3	0.0585	0.0508

**Table 4:** Values of dipole moments for a coil of the helix-like L-F PNT computed using RM1 (RHF) and Amber (after RM1) methods. All values of dipole moments are given in Debye units.

*i*	RM1 RHF				Amber			
	*Di*	*Dx*	*Dy*	*Dz*	*Di*	*Dx*	*Dy*	*Dz*
1	2.730	2.625	−0.697	0.282	2.915	2.740	−0.955	−0.282
2	3.400	2.884	0.360	−1.765	2.937	2.232	1.075	−1.578
3	2.488	1.645	1.503	1.106	2.624	0.984	1.348	2.025
4	2.615	1.710	1.869	0.650	2.671	2.086	1.603	0.463
5	2.558	−0.760	2.203	1.054	2.844	−1.325	1.618	1.928
6	2.449	−0.956	2.224	0.370	2.564	−0.554	2.429	−0.608
7	2.997	−1.096	0.851	−2.656	2.372	−1.185	−0.194	−2.046
8	2.258	−1.456	0.790	−1.534	1.631	−0.631	0.534	−1.406
9	2.436	−1.265	−1.928	−0.785	2.691	−2.137	−1.636	−0.026
10	2.611	−1.789	−1.795	0.630	1.504	−1.081	−0.999	0.310
11	1.887	−0.942	−0.771	−1.442	1.165	−1.135	−0.254	0.068
12	2.201	−0.996	−1.873	0.586	1.771	−0.819	−1.533	−0.338
*Dsum*	30.630	−0.396	3.433	−3.504	27.692	2.111	3.036	−2.138
*Dcoil*	9.995	6.624	2.820	−6.934	6.3004	4.2597	2.0522	−4.164
*Dav*	2.553				2.308			

RM1: *(Dav)^3^* = 16.640 Debye^3^; Amber: *(Dav)^3^* = 12.294 Debye^3^.

**Table 5:** Values of dipole moments for a coil of the helix-like D-F PNT computed using RM1 (RHF) and Amber (after RM1) methods. All values of dipole moments are given in Debye units.

*i*	RM1 RHF				Amber			
	*Di*	*Dx*	*Dy*	*Dz*	*Di*	*Dx*	*Dy*	*Dz*
1	2.992	−2.037	2.152	0.414	3.041	−2.109	1.870	1.142
2	3.676	−3.516	−0.872	−0.630	2.980	−2.724	−0.862	−0.846
3	2.864	−2.084	−1.822	−0.735	2.727	−1.612	−1.660	−1.443
4	2.943	0.259	−2.654	1.244	2.797	−0.790	−2.055	1.726
5	2.746	0.852	−2.590	−0.325	2.780	1.098	−2.542	0.245
6	2.821	1.415	−2.392	−0.483	3.192	1.285	−2.357	−1.726
7	3.781	1.444	−3.251	1.282	3.691	1.197	−2.894	1.953
8	2.503	1.317	2.048	0.578	2.387	2.070	1.060	−0.539
9	2.888	1.720	1.513	1.758	2.483	1.376	1.563	1.353
10	3.282	−1.128	2.525	1.766	2.732	−0.748	2.323	1.230
11	3.762	−3.239	1.798	0.657	3.072	−2.448	1.774	0.547
12	2.667	−2.229	0.793	−1.231	2.836	−2.774	0.589	0.034
*Dsum*	36.925	−7.224	−2.752	4.294	34.718	−6.179	−3.191	3.675
*Dcoil*	9.599	−6.395	−5.676	4.362	8.234	−4.333	−5.081	4.818
*Dav*	3.077				2.893			

RM1: *(Dav)*^3^ = 29.134 Debye^3^; Amber: *(Dav)**^3^*** = 24.217 Debye^3^.

**Table 6:** Magnitudes and signs of the chirality obtained for L-F and D-F PNTs for various calculating methods.

Type of PNT	L-F		D-F	
Calculating method	RM1 RHF	Amber	RM1 RHF	Amber
*c*_total_, Debye^3^	20.266	18.171	−19.647	−26.204
*c*_norm_	1.219	1.479	−0.674	−1.082
Chirality sign	positive	positive	negative	negative
Chirality symbol	D	D	L	L

## Data Availability

The data presented in this study are available on request from the corresponding author.

## Supplementary Materials

The following are available online at https://www.mdpi.com/article/10.3390/nano11123299/s1, Video S1: Assembly of a left-handed nanotube based on 48 phenylalanine D-monomers.

## Appendix A

**Table A1:** The calculated parameters of π-helices.

Protein PDB ID	Chain/Residues	Length of Helix	Chirality Value	Chirality Sign	Protein Class
1A8E	A/124-130	7	0.1150	Right-handed	Iron transport
1A8I	A/488-495	8	0.1632	Right-handed	Transferase
1BDM	A/217-223	7	0.1350	Right-handed	Oxidoreductase
1BG6	A/297-303	7	0.1373	Right-handed	Oxidoreductase
1D3G	A/37-43	7	0.1283	Right-handed	Oxidoreductase
1DK8	A/242-249	8	0.1711	Right-handed	Signaling protein
1DYS	A/112-118	7	0.1250	Right-handed	Hydrolase
1DZ4	A/150-156	7	0.1273	Right-handed	Oxidoreductase
1E3A	A/138-146	9	0.2050	Right-handed	Hydrolase
1EGU	A/292-298	7	0.1323	Right-handed	Lyase
1EGU	A/441-447	7	0.1229	Right-handed	Lyase
1EI5	A/177-183	7	0.1299	Right-handed	Hydrolase
1EK6	A/105-111	7	0.1502	Right-handed	Isomerase
1EL4	A/44-51	8	0.1665	Right-handed	Luminescent protein
1ELK	A/95-101	7	0.1421	Right-handed	Endocytosis/Exocytosis
1EOK	A/111-118	8	0.1670	Right-handed	Hydrolase
1EVY	A/257-263	7	0.1543	Right-handed	Oxidoreductase
1F3A	A/126-132	7	0.1235	Right-handed	Transferase
1F24	A/214-220	7	0.1336	Right-handed	Oxidoreductase
1FQA	A/279-285	7	0.1304	Right-handed	Sugar binding protein
1KVE	B/177-183	7	0.1271	Right-handed	Toxin
1MRO	A/313-324	12	0.2911	Right-handed	Transferase
1MUC	A/70-76	7	0.1270	Right-handed	Isomerase
1NCI	A/40-46	7	0.1125	Right-handed	Cell adhesin protein
1QGW	C/105-111	7	0.1287	Right-handed	Photosynthesis
1QH3	A/154-160	7	0.1316	Right-handed	Hydrolase
1QH8	A/63-72	10	0.2242	Right-handed	Oxidoreductase
1QLM	A/88-94	7	0.1216	Right-handed	Hydrolase
1QMG	A/349-355	7	0.1503	Right-handed	Oxidoreductase
1QMG	A/490-502	13	0.3543	Right-handed	Oxidoreductase
1SMD	A/27-33	7	0.1276	Right-handed	Hydrolase
1SUR	A/131-137	7	0.1303	Right-handed	Oxidoreductase
1THG	A/424-430	7	0.1360	Right-handed	Hydrolase
2SCP	A/56-62	7	0.1344	Right-handed	Binding protein
1B16	A/104-110	7	0.1277	Right-handed	Oxidoreductase
1B25	A/479-485	7	0.1247	Right-handed	Oxidoreductase
1BDB	A/112-118	7	0.1379	Right-handed	Oxidoreductase
1BXK	A/98-104	7	0.1439	Right-handed	Lyase
1C3P	A/97-103	7	0.1447	Right-handed	Lyase
1C3W	A/213-219	7	0.1543	Right-handed	Ion transport
1C7S	A/641-647	7	0.0941	Right-handed	Hydrolase
1C7S	A/801-807	7	0.1273	Right-handed	Hydrolase
1CB8	A/267-273	7	0.1317	Right-handed	Lyase
1COJ	A/26-33	8	0.1745	Right-handed	Oxidoreductase
1CXP	C/291-297	7	0.1234	Right-handed	Oxidoreductase
1CYD	A/104-110	7	0.1336	Right-handed	Oxidoreductase
1D3Y	A/253-259	7	0.1317	Right-handed	Isomerase
1D8D	A/343-349	7	0.1146	Right-handed	Transferase
1DC1	A/98-104	7	0.1341	Right-handed	Hydrolase
1DEK	A/137-145	9	0.1858	Right-handed	Transferase
1DJ0	A/81-87	7	0.1546	Right-handed	Lyase
1DOZ	A/265-274	10	0.2409	Right-handed	Lyase
1DQA	A/733-740	8	0.1738	Right-handed	Oxidoreductase
1DQS	A/142-148	7	0.1262	Right-handed	Lyase
1DXR	C/277-283	7	0.1231	Right-handed	Photosynthesis
1DXR	H/27-33	7	0.1234	Right-handed	Photosynthesis
1DXR	L/129-135	7	0.1276	Right-handed	Photosynthesis
1DXR	M/156-162	7	0.1229	Right-handed	Photosynthesis
1EA5	A/396-402	7	0.1241	Right-handed	Hydrolase
1EA5	A/522-528	7	0.1313	Right-handed	Hydrolase
1EWF	A/181-187	7	0.1379	Right-handed	Antibiotic
1EYZ	A/119-125	7	0.1426	Right-handed	Transferase
1F24	A/140-146	7	0.1259	Right-handed	Oxidoreductase
1FDS	A/111-117	7	0.1373	Right-handed	Oxidoreductase
1FP3	A/273-279	7	0.1241	Right-handed	Isomerase
1FRP	A/276-282	7	0.1304	Right-handed	Hydrolase
1FSW	A/174-180	7	0.1318	Right-handed	Hydrolase
1FUR	A/155-161	7	0.1348	Right-handed	Hydrolase
1FUR	A/383-389	7	0.1302	Right-handed	Hydrolase
1G8K	A/181-187	7	0.1299	Right-handed	Oxidoreductase
1G8K	A/242-248	7	0.1418	Right-handed	Oxidoreductase
1GAI	A/150-156	7	0.1346	Right-handed	Hydrolase
1HFE	S/71-77	7	0.1228	Right-handed	Oxidoreductase
1HVB	A/183-189	7	0.1282	Right-handed	Hydrolase
1I0H	A/26-32	7	0.1402	Right-handed	Oxidoreductase
1LML	A/155-161	7	0.1243	Right-handed	Hydrolase
1LST	A/126-132	7	0.1449	Right-handed	Amino-acid binding protein
1LST	A/165-171	7	0.1207	Right-handed	Amino-acid binding protein
1MTY	B/140-150	11	0.2786	Right-handed	Oxidoreductase
1MTY	B/297-304	8	0.1463	Right-handed	Oxidoreductase
1MTY	D/185-191	7	0.1139	Right-handed	Oxidoreductase
1MTY	D/202-214	13	0.3008	Right-handed	Oxidoreductase
1MTY	D/306-318	13	0.3226	Right-handed	Oxidoreductase
1MTY	D/379-385	7	0.1590	Right-handed	Oxidoreductase
1ONE	A/67-73	7	0.1322	Right-handed	Lyase
1PHN	A/107-113	7	0.1335	Right-handed	Electron transport
1QOY	A/26-32	7	0.1315	Right-handed	Toxin
1SVF	A/171-177	7	0.1381	Right-handed	Viral protein
1UOK	A/393-399	7	0.1330	Right-handed	Hydrolase
1YAC	A/114-120	7	0.1410	Right-handed	Hydrolase
1YGE	A/261-267	7	0.1353	Right-handed	Oxidoreductase
1YGE	A/494-506	13	0.3152	Right-handed	Oxidoreductase
1YGE	A/684-690	7	0.1398	Right-handed	Oxidoreductase
2EBN	A/257-263	7	0.1327	Right-handed	Hydrolase
2HMQ	A/101-107	7	0.1429	Right-handed	Oxygen transport
2OLB	A/301-308	8	0.1504	Right-handed	Binding protein
4PAN	A/325-331	7	0.1580	Right-handed	Signaling protein
5CSM	A/233-239	7	0.1380	Right-handed	Isomerase
7A3H	A/146-152	7	0.1293	Right-handed	Hydrolase
9GAF	A/186-192	7	0.1005	Right-handed	Hydrolase

**Table A2:** The calculated parameters of α-turns.

Family or Group	Protein PDB ID	Chain/Residues	Chirality Value	Chirality Sign	Protein Class
F1	1AAP	A/24-28	0.0917	Right-handed	Proteinase Inhibitor (Trypsin)
	1ACX	A/82-86	0.0735	Right-handed	Antibacterial Protein
	2AK3	A/149-153	0.0694	Right-handed	Transferase (Phosphotransferase)
	3COX	A/391-395	0.0852	Right-handed	Oxidoreductase (Oxygen Receptor)
	1DRF	A/152-156	0.0837	Right-handed	Oxidoreductase (Ch-Nh(D)-Nad Or Nadp (A))
	1ECA	A/38-42	0.0884	Right-handed	Oxygen Transport
	1GD1	O/47-51	0.0668	Right-handed	Oxidoreductase (Aldehyde(D)-Nad (A))
	1GOX	A/345-349	0.1173	Right-handed	Oxidoreductase (Oxygen(A))
	1MBA	A/43-47	0.0861	Right-handed	Oxygen Storage
	1OMD	A/2-6	0.0911	Right-handed	Calcium Binding Protein
	1OVA	A/277-281	0.0764	Right-handed	Serpin
	1OVA	A/318-322	0.1015	Right-handed	Serpin
	1RDG	A/6-10	0.0552	Right-handed	Electron Transfer (Iron-Sulfur Protein)
	1RDG	A/14-18	0.0976	Right-handed	Electron Transfer (Iron-Sulfur Protein)
	1RDG	A/39-43	0.0585	Right-handed	Electron Transfer (Iron-Sulfur Protein)
	2SN3	A/7-11	0.0626	Right-handed	Toxin
	2SN4	A/31-35	0.0847	Right-handed	Toxin
	1THB	A/113-117	0.0937	Right-handed	Oxygen Transport
	2ACT	A/85-89	0.1009	Right-handed	Hydrolase (Proteinase)
	2AZA	A/40-44	0.0952	Right-handed	Electron Transport Protein (Cuproprotein)
	2CA2	A/34-38	0.1028	Right-handed	Lyase (Oxo-Acid)
	2CPP	A/77-81	0.0883	Right-handed	Oxidoreductase (Oxygenase)
	2CPP	A/328-332	0.1000	Right-handed	Oxidoreductase (Oxygenase)
	2CSC	A/59-63	0.0674	Right-handed	Lyase
	2CYP	A/58-62	0.0673	Right-handed	Oxidoreductase (H2O2(A))
	2ER7	E/240-244	0.0926	Right-handed	Hydrolase/Hydrolase Inhibitor
	2FCR	A/94-98	0.0923	Right-handed	Electron Transport
	2FCR	A/148-152	0.0841	Right-handed	Electron Transport
	2LTN	A/54-58	0.0776	Right-handed	Lectin
	2LTN	A/125-129	0.0523	Right-handed	Lectin
	2LTN	A/167-171	0.0852	Right-handed	Lectin
	2RHE	A/93-97	0.0986	Right-handed	Immunoglobulin
	2RSP	A/46-50	0.1031	Right-handed	Hydrolase (Aspartyl Proteinase)
	2RSP	A/219-223	0.0798	Right-handed	Hydrolase (Serine Proteinase)
	2TRX	A/59-63	0.0993	Right-handed	Electron Transport
	3BLM	A/50-54	0.0742	Right-handed	Hydrolase
	3CLA	A/97-101	0.0833	Right-handed	Transferase (Acyltransferase)
	3CLA	A/194-198	0.0880	Right-handed	Transferase (Acyltransferase)
	4FGF	A/67-71	0.0920	Right-handed	Growth Factor
	3GRS	A/164-168	0.0968	Right-handed	Oxidoreductase (Flavoenzyme)
	4ENL	A/102-106	0.0738	Right-handed	Carbon-Oxygen Lyase
	5FD1	A/35-39	0.0986	Right-handed	Electron Transport(Iron-Sulfur)
	5CPA	A/3-7	0.1063	Right-handed	Hydrolase (C-Terminal Peptidase)
	5CPA	A/29-33	0.0905	Right-handed	Hydrolase (C-Terminal Peptidase)
	5P21	A/145-149	0.0840	Right-handed	Oncogene Protein
	6LDH	A/181-185	0.1114	Right-handed	Oxidoreductase(Choh(D)-Nad(A))
F2	2AK3	A/137-141	0.0723	Right-handed	Transferase (Phosphotransferase)
	3COX	A/453-457	0.0649	Right-handed	Oxidoreductase (Oxygen Receptor)
	1FKF	A/87-91	0.0400	Right-handed	Isomerase
	1GD1	O/129-133	0.0791	Right-handed	Oxidoreductase (Aldehyde(D)-Nad(A))
	1GD2	O/267-271	0.0761	Right-handed	Oxidoreductase (Aldehyde(D)-Nad(A))
	2TEC	E/261-265	0.0750	Right-handed	Complex(Serine Proteinase-Inhibitor)
	2TRX	A/49-53	0.0799	Right-handed	Electron Transport
	5CPA	A/89-93	0.0664	Right-handed	Hydrolase (C-Terminal Peptidase)
g1	4GCR	A/47-51	−0.0291	Left-handed	Eye Lens Protein
	4GCR	A/136-140	−0.0283	Left-handed	Eye Lens Protein
	2CYP	A/35-39	−0.0422	Left-handed	Oxidoreductase (H2O2(A))
	2FBJ	A/48-52	−0.0411	Left-handed	Immunoglobulin
	2PRK	A/212-216	−0.0230	Left-handed	Serine Proteinase
g2	2FBJ	H/100-104	−0.0355	Left-handed	Immunoglobulin
	2RHE	A/50-54	−0.0478	Left-handed	Immunoglobulin
g3	1FKF	A/82-86	0.0384	Right-handed	Isomerase
	1OVA	A/69-73	0.0523	Right-handed	Serpin
	3GRS	A/55-59	0.0351	Right-handed	Oxidoreductase (Flavoenzyme)
	4BP2	A/25-29	−0.0123	Left-handed	Carboxylic Ester Hydrolase Zymogen
g4	2ACT	A/188-192	−0.0636	Left-handed	Hydrolase (Proteinase)
	2LZM	A/27-31	−0.0613	Left-handed	Hydrolase (O-Glycosyl)
	3APR	E/11-15	−0.0684	Left-handed	Hydrolase/Hydrolase Inhibitor
	2FOX	A/56-60	−0.0741	Left-handed	Electron Transport
g5	1FX1	A/71-75	0.1042	Right-handed	Electron Transfer (Flavoprotein)
	1YPI	A/25-29	0.1020	Right-handed	Isomerase (Intramolecular Oxidoreductase)
	2FOX	A/40-44	0.1004	Right-handed	Electron Transport
g6	1GD1	O/300-304	−0.0084	Left-handed	Oxidoreductase (Aldehyde(D)-Nad(A))
	2LTN	A/100-104	−0.0026	Left-handed	Lectin
	4PEP	A/9-13	−0.0003	Left-handed	Hydrolase (Acid Proteinase)
g7	2AK3	A/129-133	0.0784	Right-handed	Transferase (Phosphotransferase)
	1FX1	A/72-76	0.1092	Right-handed	Electron Transfer (Flavoprotein)
Other	1RBP	A/63-67	−0.0099	Left-handed	Retinol Transport
	2FBJ	L/166-170	0.0745	Right-handed	Immunoglobulin

**Table A3:** The calculated parameters of Ω-loops.

Protein PDB ID	Chain/Residues	Length of Loop	Chirality Value	Chirality Sign	Protein Class
1ABE	A/93-99	7	−0.0280	Left-handed	Binding Protein
1ABE	A/142-148	7	0.1496	Right-handed	Binding Protein
1ABE	A/203-208	6	0.0231	Right-handed	Binding Protein
1ABE	A/236-248	13	0.1632	Right-handed	Binding Protein
1ABE	A/289-294	6	0.0232	Right-handed	Binding Protein
1ABE	A/299-304	6	0.0464	Right-handed	Binding Protein
2ACT	A/8-13	6	0.0508	Right-handed	Hydrolase (Proteinase)
2ACT	A/58-64	7	0.0462	Right-handed	Hydrolase (Proteinase)
2ACT	A/89-103	15	−0.1453	Left-handed	Hydrolase (Proteinase)
2ACT	A/139-144	6	0.0709	Right-handed	Hydrolase (Proteinase)
2ACT	A/141-156	16	0.1703	Right-handed	Hydrolase (Proteinase)
2ACT	A/182-192	11	0.0027	Right-handed	Hydrolase (Proteinase)
2ACT	A/198-205	8	−0.0151	Left-handed	Hydrolase (Proteinase)
2ACT	A/203-209	7	−0.0605	Left-handed	Hydrolase (Proteinase)
8ADH	A/14-21	8	−0.0071	Left-handed	Oxidoreductase (Nad(A)-Choh(D))
8ADH	A/100-112	13	0.3005	Right-handed	Oxidoreductase (Nad(A)-Choh(D))
8ADH	A/115-122	8	−0.0038	Left-handed	Oxidoreductase (Nad(A)-Choh(D))
8ADH	A/122-128	7	−0.0191	Left-handed	Oxidoreductase (Nad(A)-Choh(D))
8ADH	A/282-287	6	0.0022	Right-handed	Oxidoreductase (Nad(A)-Choh(D))
3ADK	A/133-142	10	0.0617	Right-handed	Transferase(Phosphotransferase)
2ALP	A/217-224	8	−0.0460	Left-handed	Hydrolase (Serine Proteinase)
3APP	A/41-55	15	0.2606	Right-handed	Hydrolase (Acid Proteinase)
3APP	A/129-136	8	0.0022	Right-handed	Hydrolase (Acid Proteinase)
3APP	A/139-149	11	0.4213	Right-handed	Hydrolase (Acid Proteinase)
3APP	A/184-192	9	0.0463	Right-handed	Hydrolase (Acid Proteinase)
2APR	A/8-17	10	−0.1572	Left-handed	Hydrolase (Aspartic Proteinase)
2APR	A/18-31	14	−0.2057	Left-handed	Hydrolase (Aspartic Proteinase)
2APR	A/43-58	16	0.0879	Right-handed	Hydrolase (Aspartic Proteinase)
2APR	A/61-69	9	0.1019	Right-handed	Hydrolase (Aspartic Proteinase)
2APR	A/76-83	8	−0.0071	Left-handed	Hydrolase (Aspartic Proteinase)
2APR	A/90-103	14	−0.1480	Left-handed	Hydrolase (Aspartic Proteinase)
2APR	A/129-138	10	0.0383	Right-handed	Hydrolase (Aspartic Proteinase)
2APR	A/189-197	9	0.0337	Right-handed	Hydrolase (Aspartic Proteinase)
2APR	A/203-211	9	−0.0328	Left-handed	Hydrolase (Aspartic Proteinase)
2APR	A/216-226	11	0.0677	Right-handed	Hydrolase (Aspartic Proteinase)
2APR	A/227-232	6	0.1507	Right-handed	Hydrolase (Aspartic Proteinase)
2APR	A/233-248	16	0.0514	Right-handed	Hydrolase (Aspartic Proteinase)
2APR	A/243-250	8	−0.0071	Left-handed	Hydrolase (Aspartic Proteinase)
2APR	A/261-273	13	−0.1703	Left-handed	Hydrolase (Aspartic Proteinase)
2APR	A/280-287	8	−0.0761	Left-handed	Hydrolase (Aspartic Proteinase)
2APR	A/291-299	9	−0.0724	Left-handed	Hydrolase (Aspartic Proteinase)
1AZU	A/9-15	7	−0.0069	Left-handed	Electron Transport (Copper Binding)
1AZU	A/35-46	12	0.0569	Right-handed	Electron Transport (Copper Binding)
1AZU	A/67-72	6	0.0376	Right-handed	Electron Transport (Copper Binding)
1AZU	A/73-83	11	0.0646	Right-handed	Electron Transport (Copper Binding)
1AZU	A/84-92	9	−0.0023	Left-handed	Electron Transport (Copper Binding)
1AZU	A/112-118	7	0.0420	Right-handed	Electron Transport (Copper Binding)
1CYO	A/32-47	16	0.4146	Right-handed	Electron Transport
1BP2	A/23-30	8	0.0360	Right-handed	Hydrolase
1BP3	A/25-39	15	−0.0675	Left-handed	Hydrolase
1BP4	A/56-66	11	0.2318	Right-handed	Hydrolase
2BP2	A/23-30	8	0.0360	Right-handed	Hydrolase Zymogen
2BP3	A/25-39	15	−0.0675	Left-handed	Hydrolase Zymogen
2BP4	A/61-68	8	−0.0056	Left-handed	Hydrolase Zymogen
256B	A/16-25	10	0.1574	Right-handed	Electron Transport
256B	A/47-58	12	0.1626	Right-handed	Electron Transport
351C	A/16-25	10	−0.0117	Left-handed	Electron Transport
351C	A/51-62	12	0.0243	Right-handed	Electron Transport
155C	A/21-28	8	−0.1010	Left-handed	Electron Transport
155C	A/47-54	8	0.0381	Right-handed	Electron Transport
155C	A/83-95	13	0.3003	Right-handed	Electron Transport
155C	A/128-133	6	0.0115	Right-handed	Electron Transport
2C2C	A/18-33	16	−0.2105	Left-handed	Electron Transport Protein (Cytochrome)
2C2C	A/30-43	14	0.1534	Right-handed	Electron Transport Protein (Cytochrome)
2C2C	A/41-56	16	0.2603	Right-handed	Electron Transport Protein (Cytochrome)
2C2C	A/74-89	16	0.4033	Right-handed	Electron Transport Protein (Cytochrome)
2CAB	A/6-12	7	0.0716	Right-handed	Hydro-Lyase
2CAB	A/17-24	8	0.1911	Right-handed	Hydro-Lyase
2CAB	A/78-87	10	0.1359	Right-handed	Hydro-Lyase
2CAB	A/98-104	7	0.0950	Right-handed	Hydro-Lyase
2CAB	A/108-114	7	−0.0536	Left-handed	Hydro-Lyase
2CAB	A/128-140	13	0.2908	Right-handed	Hydro-Lyase
2CAB	A/197-204	8	−0.0225	Left-handed	Hydro-Lyase
2CAB	A/230-240	11	−0.0683	Left-handed	Hydro-Lyase
1CA2	A/5-16	12	0.1204	Right-handed	Lyase (Oxo-Acid)
1CA3	A/17-23	7	0.1274	Right-handed	Lyase (Oxo-Acid)
1CA4	A/98-103	6	0.0470	Right-handed	Lyase (Oxo-Acid)
1CA5	A/108-114	7	−0.0629	Left-handed	Lyase (Oxo-Acid)
1CA6	A/128-140	13	0.3094	Right-handed	Lyase (Oxo-Acid)
1CA7	A/166-172	7	0.0573	Right-handed	Lyase (Oxo-Acid)
1CA8	A/197-204	8	−0.0111	Left-handed	Lyase (Oxo-Acid)
1CA9	A/232-239	8	−0.0806	Left-handed	Lyase (Oxo-Acid)
2CHA	B/70-78	9	0.0557	Right-handed	Hydrolase (Serine Proteinase)
2CHA	B/94-102	9	0.0806	Right-handed	Hydrolase (Serine Proteinase)
2CHA	B/114-119	6	0.0369	Right-handed	Hydrolase (Serine Proteinase)
2CHA	C/217-224	8	−0.0060	Left-handed	Hydrolase (Serine Proteinase)
3CNA	A/13-21	9	0.0381	Right-handed	Lectin (Agglutinin)
3CNA	A/97-104	8	−0.0102	Left-handed	Lectin (Agglutinin)
3CNA	A/116-123	8	0.0756	Right-handed	Lectin (Agglutinin)
3CNA	A/147-155	9	0.0144	Right-handed	Lectin (Agglutinin)
3CNA	A/160-165	6	0.0127	Right-handed	Lectin (Agglutinin)
3CNA	A/199-209	11	0.0003	Right-handed	Lectin (Agglutinin)
3CNA	A/222-235	14	0.1464	Right-handed	Lectin (Agglutinin)
3CNA	A/229-237	9	0.0108	Right-handed	Lectin (Agglutinin)
3CPA	A/128-141	14	0.0767	Right-handed	Hydrolase (C-Terminal Peptidase)
3CPA	A/142-156	15	0.2551	Right-handed	Hydrolase (C-Terminal Peptidase)
3CPA	A/156-166	11	0.1575	Right-handed	Hydrolase (C-Terminal Peptidase)
3CPA	A/205-213	9	0.0898	Right-handed	Hydrolase (C-Terminal Peptidase)
3CPA	A/231-237	7	−0.0073	Left-handed	Hydrolase (C-Terminal Peptidase)
3CPA	A/244-250	7	−0.0284	Left-handed	Hydrolase (C-Terminal Peptidase)
3CPA	A/272-285	14	−0.0102	Left-handed	Hydrolase (C-Terminal Peptidase)
5CPV	A/18-23	6	0.0092	Right-handed	Calcium Binding
5CPV	A/64-77	14	0.2505	Right-handed	Calcium Binding
1CRN	A/33-44	12	−0.0524	Left-handed	Plant Protein
1CTX	A/1-15	15	0.0597	Right-handed	Toxin
1CTX	A/26-35	10	0.1348	Right-handed	Toxin
1CYC	A/18-32	15	−0.1967	Left-handed	Electron Transport
1CYC	A/30-43	14	0.0486	Right-handed	Electron Transport
1CYC	A/40-54	15	0.1398	Right-handed	Electron Transport
1CYC	A/70-84	15	0.1522	Right-handed	Electron Transport
3CYT	O/18-32	15	−0.1637	Left-handed	Electron Transport (Heme Protein)
3CYT	O/34-43	10	0.1153	Right-handed	Electron Transport (Heme Protein)
3CYT	O/40-54	15	0.1250	Right-handed	Electron Transport (Heme Protein)
3CYT	O/70-84	15	0.2509	Right-handed	Electron Transport (Heme Protein)
1ECD	A/33-42	10	0.3042	Right-handed	Oxygen Transport
1ECD	A/41-49	9	0.0899	Right-handed	Oxygen Transport
1EST	A/69-80	12	0.0984	Right-handed	Hydrolase
1EST	A/94-104	11	0.1166	Right-handed	Hydrolase
1EST	A/112-118	7	−0.0091	Left-handed	Hydrolase
1EST	A/165-178	14	0.3179	Right-handed	Hydrolase
1EST	A/216-226	11	0.0211	Right-handed	Hydrolase
7FAB	L/24-29	6	0.0311	Right-handed	Immune System
7FAB	L/122-132	11	−0.0979	Left-handed	Immune System
7FAB	L/168-173	6	−0.0200	Left-handed	Immune System
7FAB	L/182-187	6	0.0160	Right-handed	Immune System
7FAB	H/72-77	6	0.0406	Right-handed	Immune System
7FAB	H/99-105	7	−0.0067	Left-handed	Immune System
1DUR	A/12-23	12	0.2808	Right-handed	Electron Transport
1DUR	A/30-41	12	0.0120	Right-handed	Electron Transport
1DUR	A/39-50	12	0.2652	Right-handed	Electron Transport
5NLL	A/54-61	8	−0.0590	Left-handed	Electron Transport
2GCH	F/70-78	9	0.1866	Right-handed	Hydrolase (Serine Proteinase)
2GCH	F/94-101	8	0.0378	Right-handed	Hydrolase (Serine Proteinase)
2GCH	F/112-118	7	−0.0220	Left-handed	Hydrolase (Serine Proteinase)
2GCH	G/165-176	12	0.3527	Right-handed	Hydrolase (Serine Proteinase)
2GCH	G/217-224	8	−0.0086	Left-handed	Hydrolase (Serine Proteinase)
1GPD	G/47-52	6	0.0284	Right-handed	Oxidoreductase
1GPD	G/76-82	7	0.0872	Right-handed	Oxidoreductase
1GPD	G/121-129	9	0.0052	Right-handed	Oxidoreductase
1GPD	G/183-198	16	−0.1486	Left-handed	Oxidoreductase
3GRS	A/83-89	7	−0.0102	Left-handed	Oxidoreductase (Flavoenzyme)
3GRS	A/139-147	9	−0.0760	Left-handed	Oxidoreductase (Flavoenzyme)
3GRS	A/162-172	11	0.0576	Right-handed	Oxidoreductase (Flavoenzyme)
3GRS	A/239-245	7	−0.0094	Left-handed	Oxidoreductase (Flavoenzyme)
3GRS	A/256-261	6	−0.0633	Left-handed	Oxidoreductase (Flavoenzyme)
3GRS	A/268-274	7	−0.0219	Left-handed	Oxidoreductase (Flavoenzyme)
3GRS	A/300-307	8	−0.0017	Left-handed	Oxidoreductase (Flavoenzyme)
3GRS	A/315-320	6	−0.0687	Left-handed	Oxidoreductase (Flavoenzyme)
3GRS	A/331-337	7	0.0505	Right-handed	Oxidoreductase (Flavoenzyme)
3GRS	A/404-415	12	0.2396	Right-handed	Oxidoreductase (Flavoenzyme)
3GRS	A/465-472	8	0.0467	Right-handed	Oxidoreductase (Flavoenzyme)
1HIP	A/20-26	7	0.1417	Right-handed	Electron Transfer (Iron-Sulfur Protein)
1HIP	A/28-41	14	0.0932	Right-handed	Electron Transfer (Iron-Sulfur Protein)
1HIP	A/43-49	7	0.0998	Right-handed	Electron Transfer (Iron-Sulfur Protein)
1HIP	A/44-59	16	0.1820	Right-handed	Electron Transfer (Iron-Sulfur Protein)
6LDH	A/173-188	16	0.2820	Right-handed	Oxidoreductase (Choh(D)-Nad(A))
6LDH	A/192-200	9	0.0461	Right-handed	Oxidoreductase (Choh(D)-Nad(A))
6LDH	A/203-218	16	0.0187	Right-handed	Oxidoreductase (Choh(D)-Nad(A))
6LDH	A/212-225	14	0.1010	Right-handed	Oxidoreductase (Choh(D)-Nad(A))
6LDH	A/219-226	8	0.1033	Right-handed	Oxidoreductase (Choh(D)-Nad(A))
6LDH	A/239-246	8	−0.0174	Left-handed	Oxidoreductase (Choh(D)-Nad(A))
6LDH	A/275-285	11	0.0584	Right-handed	Oxidoreductase (Choh(D)-Nad(A))
1LH1	A/41-53	13	0.3229	Right-handed	Oxygen Transport
1LH2	A/47-54	8	0.0285	Right-handed	Oxygen Transport
2LHB	A/46-59	14	0.5669	Right-handed	Oxygen Transport
2LHB	A/55-64	10	0.2227	Right-handed	Oxygen Transport
7LYZ	A/18-25	8	−0.0331	Left-handed	Hydrolase (O-Glycosyl)
7LYZ	A/36-42	7	0.0318	Right-handed	Hydrolase (O-Glycosyl)
7LYZ	A/44-52	9	−0.0407	Left-handed	Hydrolase (O-Glycosyl)
7LYZ	A/60-75	16	0.1404	Right-handed	Hydrolase (O-Glycosyl)
2LZM	A/134-139	6	0.0657	Right-handed	Hydrolase (O-Glycosyl)
1MBN	A/40-47	8	0.2021	Right-handed	Oxygen Storage
1MBS	A/37-50	14	0.5199	Right-handed	Oxygen Transport
1MBS	A/49-54	6	0.0358	Right-handed	Oxygen Transport
1MBS	A/78-84	7	0.1077	Right-handed	Oxygen Transport
2MHB	A/40-48	9	0.2250	Right-handed	Oxygen Transport
2MHB	B/39-54	16	0.4710	Right-handed	Oxygen Transport
2MHB	B/47-57	11	0.2937	Right-handed	Oxygen Transport
1NXB	A/6-13	8	−0.1576	Left-handed	Neurotoxin (Post-Synaptic)
2PAB	A/49-54	6	−0.0652	Left-handed	Transport (Thyroxine,Retinol) In Serum
9PAP	A/8-13	6	0.0535	Right-handed	Hydrolase (Sulfhydryl Proteinase)
9PAP	A/60-67	8	−0.0304	Left-handed	Hydrolase (Sulfhydryl Proteinase)
9PAP	A/86-100	15	0.0073	Right-handed	Hydrolase (Sulfhydryl Proteinase)
9PAP	A/138-153	14	0.1452	Right-handed	Hydrolase (Sulfhydryl Proteinase)
9PAP	A/175-185	11	0.0051	Right-handed	Hydrolase (Sulfhydryl Proteinase)
9PAP	A/191-198	8	0.0624	Right-handed	Hydrolase (Sulfhydryl Proteinase)
9PAP	A/198-203	6	−0.0338	Left-handed	Hydrolase (Sulfhydryl Proteinase)
1PLC	A/6-13	8	−0.0920	Left-handed	Electron Transport
1PLC	A/41-55	15	0.1393	Right-handed	Electron Transport
1PLC	A/63-68	6	0.0171	Right-handed	Electron Transport
1PLC	A/84-92	9	0.1941	Right-handed	Electron Transport
